# Random peptide mixtures entrapped within a copper-cuprite matrix: new antimicrobial agent against methicillin-resistant *Staphylococcus aureus*

**DOI:** 10.1038/s41598-019-47315-0

**Published:** 2019-08-02

**Authors:** Tal Stern Bauer, Barak Menagen, David Avnir, Zvi Hayouka

**Affiliations:** 1 Institute of Biochemistry, Food Science and Nutrition, The Hebrew University of Jerusalem, Rehovot, 76100 Israel; 2 Institute of Chemistry, The Hebrew University of Jerusalem, Jerusalem, 91904 Israel; 3 The Center for Nanoscience and Nanotechnology, The Hebrew University of Jerusalem, Jerusalem, 91904 Israel

**Keywords:** Chemical biology, Bacterial infection

## Abstract

The emergence of global antibiotic resistance necessitates the urgent need to develop new and effective antimicrobial agents. Combination of two antimicrobial agents can potentially improve antimicrobial potency and mitigate the development of resistance. Therefore, we have utilized metal molecular doping methodology whereby antimicrobial random peptides mixture (RPMs) are entrapped in a bactericidal copper metal matrix. The copper/RPM composite exhibits greater antimicrobial activity toward methicillin-resistant *Staphylococcus aureus* (MRSA) than either copper or RPMs alone. Our findings indicate that this bactericidal antimicrobial biomaterial could be utilized to efficiently eradicate antibiotic-resistant pathogenic bacteria for health, agricultural and environmental applications.

## Introduction

Bacterial resistance to antibiotics is a global concern and there is a continuing need to the develop new antimicrobial agents. Resistant bacteria in both hospitals and the environment cause prolonged hospitalization and the death of tens of thousands of people annually, worldwide. For example, methicillin-resistant *Staphylococcus aureus* (MRSA) claims the lives of approximately 19,000 people per annum in the United States alone^1^. Current solutions to this global crisis includes the use of new classes of antimicrobial agents and traditional bactericides^1^. The combination of two antimicrobial agents that possess different modes of action has been suggested as a more effective solution to eradicate resistant bacteria than using just one^2,3^.

Low concentrations of metals such as copper and silver exhibit bactericidal properties and are thought to work either extracellularly by binding and inactivating membrane proteins or intracellularly after transport into the cell^4,5^. In fact, copper has been used in ancient Egypt as early as 2000 B.C. to sterilize water and wounds^6^. Although silver has greater bactericidal activity, copper is less expensive and therefore utilized extensively in both the health and agricultural sectors^7,8^. For example, it has been used as an additive in water treatment facilities, as a disinfectant in the food industry, for hospital sterilization, to incorporate antifouling properties in paint and for wound healing^6,9^. Copper is also commonly used on a large scale in the agricultural sector for crop protection^6,10^. The microbial toxicity of copper is mediated through multifactorial pathways, one of which exploits its ability to act as a catalyst to generate reactive oxygen species (ROS), which cause damage to vital cell constituents such as proteins, lipids and DNA^5,7,11^. Copper ions can also deactivate intracellular and membrane proteins by direct interaction or via competition for the binding sites of essential metals. In both cases, inhibition or deactivation of the protein is the result of conformational changes in the protein structure^6,7^. Unfortunately, copper-resistant bacterial strains have emerged and are responsible for significant reductions in crop yields^9,12^, therefore, there is an urgent need for novel solutions to combat these resistant pathogens^5^. This has spurred study of its antimicrobial or anti-biofilm activity together with combinations of amino acids^13^, organic acids^14^, hepcidin^15^ or copper binding peptides^16,17^. Here, we present a new combination of antimicrobial agents: a copper matrix doped with antimicrobial peptides.

Antimicrobial random peptide mixtures (RPMs)^18^ were originally inspired by natural antimicrobial peptides (AMPs), which are produced by eukaryotes as part of their innate immune response to bacterial infection^19,20^. AMPs are typically cationic and act primarily via electrostatic interactions with the anionic bacterial membrane, followed by insertion whereby the hydrophobic residues cause membrane disruption and bacterial cell death. Despite the broad structural diversity of natural AMPs, we identified several common features, which we incorporated into the RPM design, to enable targeting of bacterial cell membranes. RPMs are synthesized via solid-phase peptide synthesis (SPPS) by mixing an equimolar ratio of one hydrophobic and one cationic amino acid to generate a mixture of peptides with the desired chain length and defined stereochemistry but with random sequences. Homochiral RPMs have been shown to disrupt synthetic lipid bilayers via a pore-like mode of action^18^ and were found to possess potent antimicrobial and anti-biofilm activity^21,22^. Their synthesis is both efficient and cost-effective and generates a cocktail of peptides, which may confer a broader spectrum of activity and potentially a lower probability of developing bacterial resistance^23^.

**Figure 1:**
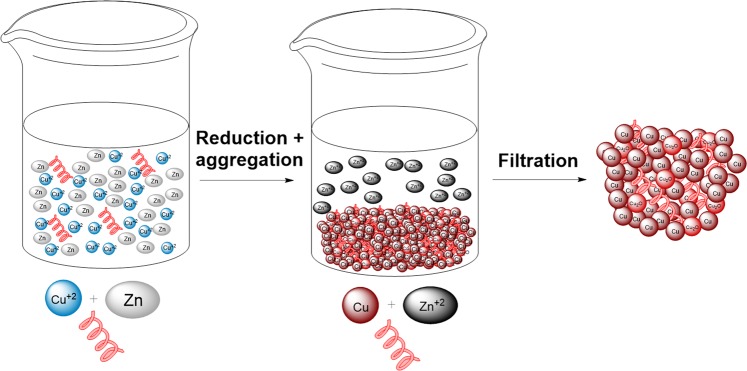
Entrapment of LK RPMs in a copper matrix. Copper ions were reduced by metallic zinc in the presence of LK RPMs to form tight aggregated copper/cuprite nanocrystals with the peptides trapped inside.

We decided to evaluate a combination treatment of antimicrobial agents, which is a strategy that has previously been successful against MRSA^3,24,25^. It has been demonstrated that the incorporation and entrapment of biologically active molecules in metals can result in synergistic activity^26^. Composites made by entrapping the antimicrobial agent chlorohexidine and silver (CH@Ag) have shown synergistic antimicrobial activity^27,28^. When the silver was replaced with copper, the composite had even greater antimicrobial activity^8^. Strong antibacterial activity was even observed by the incorporation of anti-inflammatory agents within silver^29^. Based on previous work that demonstrated the entrapment of proteins in silver^30^ and Nafion™ in copper^31^, we have developed a protocol to entrap antimicrobial RPMs in copper (Fig. 1). We selected a leucine/lysine (LK) RPM for entrapment within copper since this mixture possesses potent antibacterial activity^21,22^. The resulting composite proved to be an efficient growth inhibitor of MRSA.

## Materials and Methods

### Chemicals and bacterium

Fmoc-L-Leu-OH, Fmoc-L-Lys(Boc)-OH, 2-(1H-benzotriazole-1-yl)-1,1,3,3-tetramethyluronium hexafluorophosphate (HBTU) and rink amide resin (0.53 mmol/g) were purchased from Chem-Impex (USA). Sodium chloride, *N*,*N*-dimethylformamide (DMF), diethyl ether, trifluoroacetic acid (TFA), and *N*,*N*-diisopropylethylamine (DIEA) were purchased from Biolab, Israel. Yeast and tryptone were obtained from BD (Franklin Lakes, NJ). Copper sulfate, zinc (granular) and all other chemicals were purchased from Sigma Aldrich (Israel). The methicillin-resistant *Staphylococcus aureus* 1206 strain^32^ was obtained from Prof. B. Weisblum (UW-Madison, Wisconsin, USA).

### Peptide synthesis

RPMs were synthesized via microwave-assisted SPPS on Rink amide resin (substitution: 0.53 mmol/g, 25 μmol) in filter tubes (Silicol) as described previously^21^. Briefly, coupling reactions were conducted with binary combinations of Fmoc-L-Leu-OH and Fmoc-L-Lys (Boc)-OH. A freshly prepared stock solution containing the protected amino acids in a 1:1 molar ratio was used for each reaction. Prior to each coupling step, an aliquot containing 4 equivalents (100 μmol) of the amino acid mixture was activated with 4 equivalents of HBTU and 8 equivalents of DIEA in DMF and added to the resin. The reaction mixture was then heated to 70 °C in a MARS VI multimode microwave reactor (CEM, USA) (2 minutes ramp to 70 °C, 4 minutes hold at 70 °C) with stirring. For Fmoc deprotection we added 20% piperidine in DMF and heated the reaction solution to 80 °C (2 minutes ramp to 80 °C, 3 minutes hold at 80 °C) with stirring. After each coupling/deprotection cycle, the resin was washed 3 times with DMF. At the end of the synthesis, the peptide mixtures were cleaved from the resin by adding a solution containing 95% trifluoroacetic acid (TFA), 2.5% doubly-distilled water (DDW) and 2.5% triisopropylsilane and stirred for 3 hours. The mixture was then filtered, and the peptides precipitated by the addition of cold diethyl ether to the TFA solution and centrifuged. The supernatant was then removed and the peptide pellet dried under a stream of nitrogen, dissolved in acetonitrile/DDW and frozen with liquid nitrogen and lyophilized. The synthesis was validated by MALDI-TOF mass spectrometry. The resultant leucine and lysine peptide mixtures are referred to as the LK 10-mer and LK 20-mer RPMs, based on their chain length.

### Composite preparation

CuSO_4_ (0.200 g, 1.25 × 10^−3^ mol) was dissolved in 2.0 mL of DDW. Zinc powder (0.081 g, 1.25 × 10^−3^ mol) was added and then after 30 s of stirring, 1.0 mL of a peptide solution (0.003 g, LK 10-mer or 20-mer) was added at a ~50:1 ratio of copper to amino acid. The solution was mixed for 24 h at room temperature (~23 °C). The resulting precipitate was filtered through a sintered glass funnel (pore size of 10–16 μm), washed with 30 mL of DDW and dried overnight under vacuum. Non-doped copper composite was prepared by the same procedure without adding any peptides.

### Measurement of copper ion concentration

The copper ion concentration was measured as follows: 10 mg of the composites were dispersed in 50 mL Luria broth (LB) growth media at 37 °C and shaken at 180 rounds per minute (RPM) for 8 hours. A 5.0 mL sample was then removed from the suspension, filtered through a 0.45 µm filter and digested with 3.0 mL of 65% HNO_3_ and 2.0 mL of 30% H_2_O_2_. The samples were dissolved for analysis and measured by inductively coupled plasma mass spectrometry (ICP-MS)^33,34^.

### Growth inhibition assays

LB inoculated with MRSA was incubated overnight at 37 °C with shaking at 180 RPM. A bacterial suspension with optical density (O.D.) of 0.1 at 600 nm was then prepared and diluted 1,000-fold in LB (to approximately 10^5^ CFU/mL). The tested composites (10 mg, 200 ppm) were added to sterile 100 mL Erlenmeyer flasks and 50 mL of the MRSA bacterial suspension was added. The flasks were incubated at 37 °C with shaking at 180 RPM for 24 hours. Bacterial growth was monitored by measuring the O.D._600 nm_ of 1.0 mL samples which were removed at different time points. Flasks without composites or with LK RPMs (4.0 μg/mL) were used as negative and positive controls, respectively. The percentage of growth inhibition was calculated at each time point (T_i_) according to Eq. 1:

1 $$ \% \,Growth\,Inhibition\,({T}_{i})=100-\frac{O.D.\,treatment\,at\,{T}_{i}}{O.\,D.\,negative\,comtrol\,at\,{T}_{i}}$$

### Mixing LK RPMs and copper ions assay

MRSA cells were grown as described to prepare a bacterial suspension with O.D_600 nm_ of 0.1. Aliquots (1 mL) of the suspension were mixed with an equal volume of LB containing copper sulfate and various concentrations of LK 20-mer or 10-mer peptides to give final concentrations of 400 ppm copper sulfate with 25 or 12 μg/mL of LK 20-mer, or with 200 or 100 μg/mL of LK 10-mer. After vortex mixing, a 1 mL sample was taken for O.D. measurement and the remaining 1 mL sample was incubated for 6 hours at 37 °C, 100 RPM. After this time, the O.D. was remeasured and the percentage growth was calculated according to Eq. 2:

2 $${\rm{ \% }}\,Growth\,({T}_{i})=\frac{O.D.\,{T}_{6htreatment}-O.D.\,{T}_{0htreatment}\,}{O.D.\,{T}_{6hcontrol}-O.D.\,{T}_{0hcontrol}}\times 100$$

### Assessment of peptide release

Two methods were used to examine the release of peptides from the composites. (I) Composites (10 mg) were suspended in 50 mL DDW and incubated for 6.5 hours with shaking at 37 °C, 180 RPM. The suspension was then filtered through a sintered glass funnel (size pores of 10–16 μm). The filtered composite was then dried under vacuum and the organic element content was analyzed as described below. A lack of change in the weights of the elements indicated that the peptides were not released from the composite. (II) The filtered solution was lyophilized and then suspended with 80 μL DDW. The protein content of a 50 μL aliquot was analyzed by the addition of 450 μL of 4-fold diluted Bradford reagent. After 5 minutes of incubation in the dark, the peptide content was measured by reading the O.D. at 595 nm against a standard curve prepared with serial dilutions of free LK RPMs in DDW. The linear range was 6–100 μg/mL.

### Instrumentation

RPMs were characterized on a MALDI-TOF MS Microflex LRF (Bruker Daltonik GmbH, Germany). UV-Vis absorbance spectroscopy was carried out with Genesys UV-vis spectrophotometer (Thermo Spectronic). Elemental analysis (nitrogen, carbon, hydrogen, and sulfur) of at least 4 different batches was carried out using a Thermo Elemental Analyzer 1120. Thermogravimetric analysis (TGA) from 50 °C to 800 °C was conducted with a Mettler-Toledo TGA/SDTA 851e, at a heating rate of 10 °C per minute under N_2_. Density measurements were carried out with a Micromeritics AccuPyc 1340 instrument using helium as the displacing gas. The copper ion concentration was measured by an axial inductively coupled plasma optical emission spectrometer (ICP-OES) model ‘ARCOS’ from Spectro GMBH (Germany). Scanning electron microscope (SEM) and energy-dispersive X-ray spectroscopy (EDAX) analysis was carried out on a Sirion (FEI) high resolution (HR) SEM instrument. X-ray powder diffraction (XRD) measurements were performed on a D8 Advance diffractometer (Bruker AXS, Karlsruhe, Germany) with secondary Graphite monochromator, 2° Sollers slits and a 0.2 mm receiving slit. The powder samples were placed on low background quartz sample holders. X-ray diffraction (XRD) patterns from 5° to 85° 2θ were recorded at room temperature using CuKα radiation (λ = 0.15418 nm) under the following measurement conditions: tube voltage of 40 kV, tube current of 40 mA, step scan mode with a step size of 0.02° 2θ and a counting time of 1 s per step. The Scherrer equation was used to obtain the crystalline size from the experimental XRD data. The instrumental broadening was determined using LaB6 powder (NIST SRM 660).

## Results

LK 10-mer and 20-mer RPMs were synthesized via 9-fluorenylmethyloxycarbonyl (Fmoc) SPPS^21^. Characterization was validated by mass spectrometry (Fig. SI1). Copper was doped with the RPMs by a modified version of the heterogeneous doping methodology, which is based on the reduction of the copper cation with metallic zinc (see Fig. 1 and Material and methods section)^31^. A copper ([Cu]) control was prepared by the same procedure, but in the absence of the RPMs.

**Figure 2:**
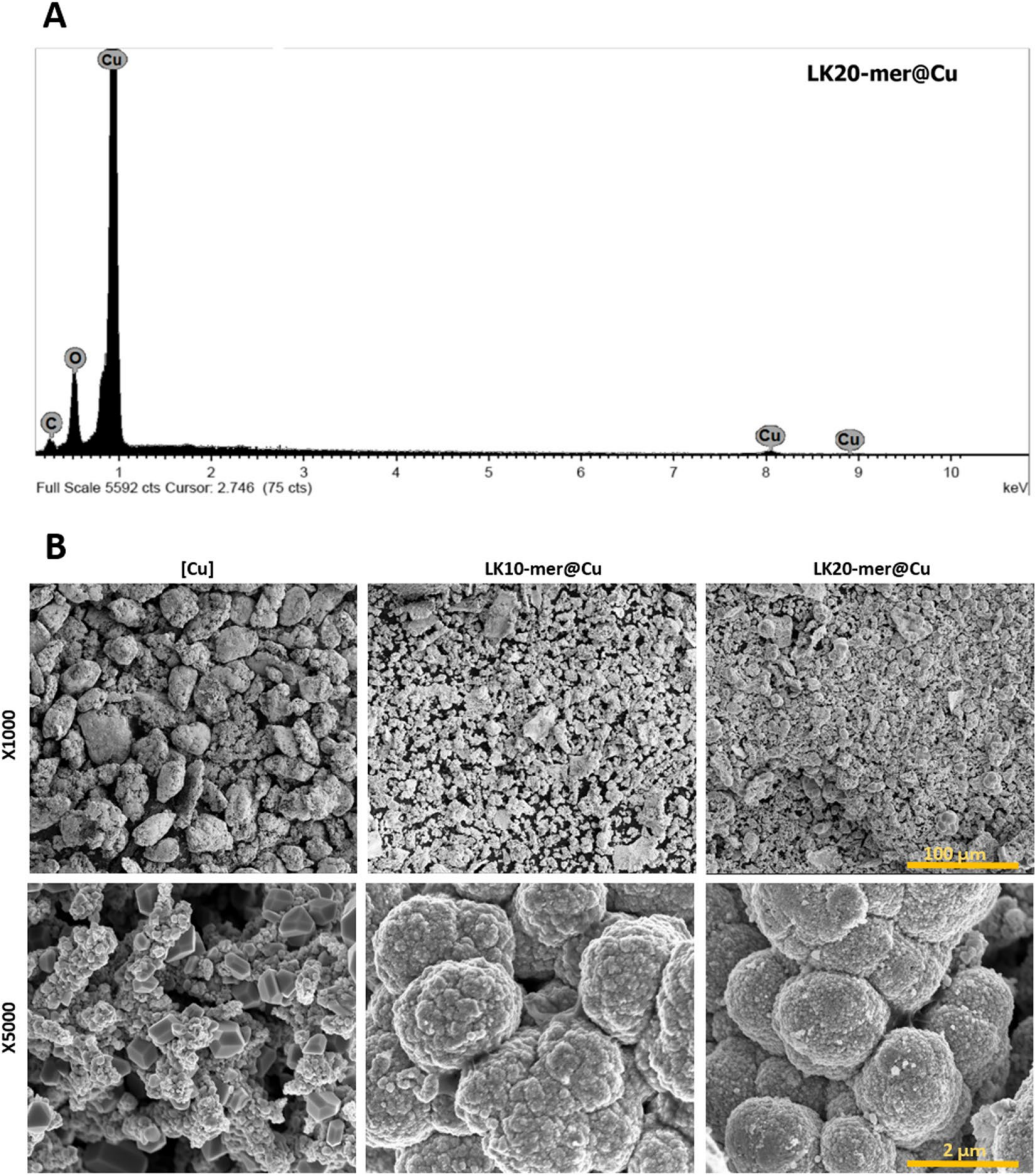
Characterizing the new composite materials. (**A)** Energy dispersive X-ray analysis of LK20-mer@[Cu]. (**B)** Structure and morphology of [Cu] composites (left column, LK20-mer@[Cu] (right column) and LK10-mer (middle column) composites.

The new materials, LK20-mer@[Cu] and LK10-mer@[Cu], were characterized by several chemical and physical measurements. The densities of the LK20-mer@[Cu] and LK10-mer@[Cu] were lower (7.2 and 7.3 g/cm^3^ respectively) than that of the non-doped copper composite (8.0 g/cm^3^) as the peptides interfere with the growth of the copper crystals during reduction. Energy dispersive X-ray analysis (Fig. 2A) showed that the composites contained only copper and organic material and no detectable traces of zinc. The use of a lower copper to zinc ratio (1:1.2) reveals residual traces of zinc (Fig. SI2), indicating that the 1:1 ratio is optimal for a contamination-free reduction. The structure and morphology of the composites were characterized by SEM (Fig. 2B), and showed nanocrystals of copper tightly aggregated to form micron-sized particles form larger clusters. Interestingly, as observed by the X1000 magnification (Fig. 2B, upper row), the peptide entrapment appears to reduce the particle size with the longer chain random peptides resulting a smaller particle. This reduction in size might be attributed to inhibition of nanocrystal growth caused by the adsorptive interactions of the LK RPM with copper.

**Figure 3:**
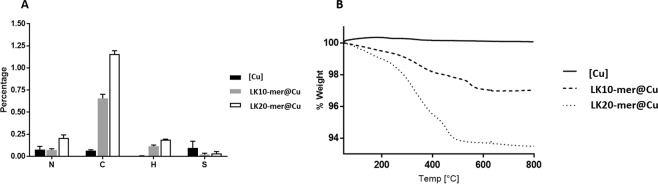
Chemical analysis of LK entrapped composites. (**A)** Elemental analysis of the designed composites presented as percentage weight. The chart shows the average and standard errors of 3 independent replicates. (**B**) Thermal gravity analysis of composites. [Cu]: smooth line; LK10-mer@[Cu]: dashed line; LK20-mer@[Cu]: dotted line.

Elemental analysis detected the presence of organic elements in the LK@[Cu] composites as opposed to the undoped copper, which confirmed entrapment of the RPMs in the copper matrix (Fig. 3A). Higher percentages of nitrogen and carbon (0.21% and 1.16%) were detected in the LK20-mer@[Cu] composites than in the LK10-mer@[Cu] composites (0.07% and 0.66%). Thermogravimetric analysis was performed to quantify the amount of entrapped organic material (Figs 3B and SI4), which measured a 2.98% weight loss for the LK10-mer@[Cu] and 6.51% for the LK20-mer@[Cu]. XRD measurements revealed that the composites contained cuprite (Cu_2_O) as well as metallic copper (Fig. SI3). The copper ([Cu]), LK10-mer@[Cu], and LK20-mer@[Cu] contained 25.8%, 33.1% and 44.1% cuprite, respectively (Fig. SI3). This result indicates that the random peptides entrapped in a copper matrix that contains both copper and cuprite, therefore the matrix termed as [Cu] along the research.

**Figure 4:**
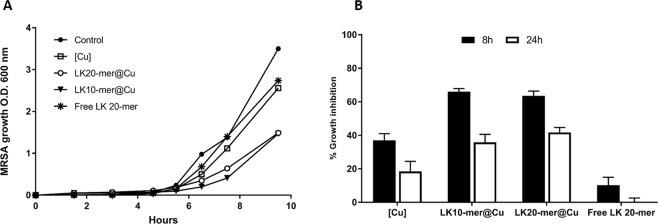
The effect of the composites on the growth of MRSA bacterial cells. (**A**) MRSA growth inhibition (from one representative experiment) in the presence of 200 ppm [Cu], LK20-mer@[Cu], LK10-mer@[Cu], 4 μg/mL free LK 20-mer and without addition (control). (**B**) The calculated average and standard errors of 7 independent repeats of growth inhibition after 8 h and 24 h.

The biological activity of each composite was then evaluated. Figure 4 shows the effect of the composites on the growth of MRSA bacterial cells. While copper ([Cu]) had only a minor effect on MRSA growth, the entrapped peptide composites at the same concentration strongly inhibited bacterial growth. This inhibition was maintained over the course of the culture, with a narrower gap after 24 hours. As presented in Fig. 4, the free LK 20-mer random peptide that was used as a control (at 4 μg/mL, in accordance with the estimated maximal amount of peptide in 200 ppm composite) did not inhibit bacterial growth.

Since the combination of both components display an enhanced antimicrobial effect, it was important to evaluate the release of copper ions from the composites. Copper ions might be released from the composites in a liquid medium according to the oligodynamic effect^35^. After 8 h incubation in growth medium, no significant differences in amount of released copper ions were detected by ICP-MS from the LK@[Cu] composites compared to the non-doped composite [Cu] (Table SI1). There was no detectable release of peptides from the composites as determined by a Bradford assay, even after extended incubation time. To verify this finding, we also performed elemental analysis of composites before and after incubation in water. The results did not show composites weight decrease, supporting the observation that there was no significant release of RPMs during incubation of the composites (Table SI2).

**Figure 5:**
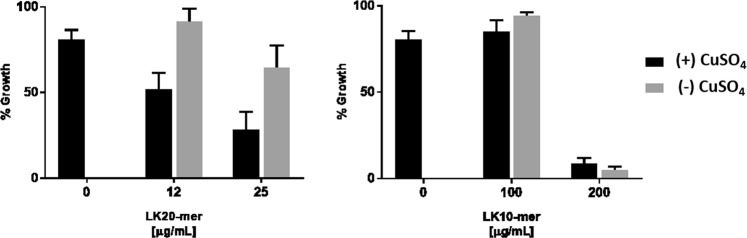
The effect of physical mixing of copper sulfate (CuSO_4_) ions and free LK RPM. Determining the antimicrobial activity by the inhibition of MRSA bacterial cells growth exhibited by physical mixing of copper sulfate (CuSO_4_) ions and free LK RPM. The results are the average and standard error of 3 independent repeats.

The antimicrobial activity of the LK@[Cu] composites was then compared with a simple mixture of the two active agents. As presented in Fig. 5, copper ions have a weak effect on the growth of MRSA (~15% lower than the control). The free LK20-mer peptide had a concentration-dependent inhibitory effect (grey bars), which was enhanced in the presence of copper ions (black bars). In contrast, the addition of copper ions to the LK10-mer did not enhance its antimicrobial activity. These findings support the notion that physical mixing of LK and copper ions is not enough to achieve the enhanced antimicrobial effect that the composites possess.

## Discussion

The LK@[Cu] composites were generated by the entrapment of random peptide mixtures in a copper-cuprite matrix represent a new material with unique properties and characteristics, rather than being simply the sum of the component parts separately. They were prepared according to the heterogeneous reduction method^31^ with zinc as the reducing agent, whereby copper agglomerates to microcrystals. RPMs interact with the copper and are entrapped within the aggregates; the zinc is then washed away as confirmed by EDAX analysis. Evidence for the formation of a new composite material is given by the reduction in particle density and size after entrapment of the peptides. This is consistent with the trend of decreasing densities of other composites (e.g. Nafion@Cu and Thionin@Cu)^31^ and may be attributable to perturbations in a typical crystal structure. The absence of detectable peptide release from the composites also suggests the formation of strong interactions between the LK RPMs and copper, in addition to their physical entrapment. This is in accord with reports that large molecules such as Nafion^31^ and the enzyme acid phosphatase^30^ are not released from similar composites, although it is possible for small molecules^8,27,29^. According to the results of the TGA and elemental analysis, the LK 20-mer RPM was entrapped more efficiently than the LK 10-mer RPM. Since copper competes with water for interaction with the peptides, we hypothesize that a longer peptide chain length, with its larger surface area, has a higher propensity to interact with the copper.

In addition to their physical properties, the composites also possessed stronger antimicrobial activity against MRSA than would be expected from the sum of its constituents. This activity is due to the unique combined effect of the two components and was not observed when the copper ions and LK 10-mer or 20-mer RPMs were tested alone or combined via physical mixing. These results indicate that the entrapment formed a new material with its own antimicrobial activity that both the copper and cuprite have a role in addition to the LK RPMs.

Interestingly, while our previous work indicated that higher concentrations of the LK 10-mer RPM was needed for growth inhibition of bacteria compared with the LK 20-mer RPM (Fig. 5) and as described at^21^, the results presented here showed that both the LK10-mer@[Cu] and LK20-mer@[Cu] composites possess similar activity. The reason for this discrepancy is not entirely clear although it probably relates to the structure of the composites. Since no peptide release from the composites was detected, we propose that the antimicrobial activity may derive from the entrapped state. In other words, the exposed 10-mer and 20-mer peptide chains within the copper matrix can interact with and disrupt bacterial cell membranes. Therefore, the LK@[Cu] composites possess an “antimicrobial surface”. In solution, copper ions are slowly released, which alters the metal matrix, thus leading to greater exposure of the bacteria to the internal RPM. According to our findings we proposed that the bacteria interacts with the composite surface then the cationic RPM attracts and disrupts the cell membrane permitting the entry of copper ions into the bacterial cells.

## Conclusion

In summary, this study describes the preparation, characterization and antimicrobial activity of LK@[Cu] composites, which are composed of copper and RPMs. These composites represent a new class of material with improved antimicrobial activity against the “superbug” MRSA. Activity towards other multidrug resistant pathogens and copper-resistant bacterial strains will require further investigation. The methodology and findings described here may be readily adapted to produce a wide variety of composites, to enable discovery of novel bactericidal agents that target resistant strains of bacteria.

## Supplementary information

Supporting information
