# Supplementary material for: Random peptide mixtures entrapped within a copper-cuprite matrix: new antimicrobial agent against methicillin-resistant Staphylococcus aureus
Source: Sci Rep. 2019 Aug 2;9:11215. doi: 10.1038/s41598-019-47315-0 (PMC6677760; doi:10.1038/s41598-019-47315-0)
Supplement: Supplementary file 1 — Supporting information [file 41598_2019_47315_MOESM1_ESM.docx]

**Supporting Information:**

**Random peptide mixtures entrapped within a copper-cuprite matrix: new antimicrobial agent against methicillin-resistant Staphylococcus aureus**

Tal Stern Bauer,^a,c^ Barak Menagen,^b,c^ David Avnir^b,c^ and Zvi Hayouka^*a,c^

^a^Institute of Biochemistry, Food Science and Nutrition, The Hebrew University of Jerusalem, Rehovot, 76100, Israel; ^b^Institute of Chemistry and ^c^The Center for Nanoscience and Nanotechnology, The Hebrew University of Jerusalem, Jerusalem 91904, Israel

* E-mail: [zvi.hayouka@mail.huji.ac.il](mailto:zvi.hayouka@mail.huji.ac.il)

**LK 20-mer**

**LK 10-mer**

**Figure SI1:** MALDI TOF MS representative spectra of leucine (L) lysine (K) random peptide mixtures after completing the synthesis. The length is controlled and the mixtures contain a pool of peptides that share a similar molecular weight but have slightly different sequences.


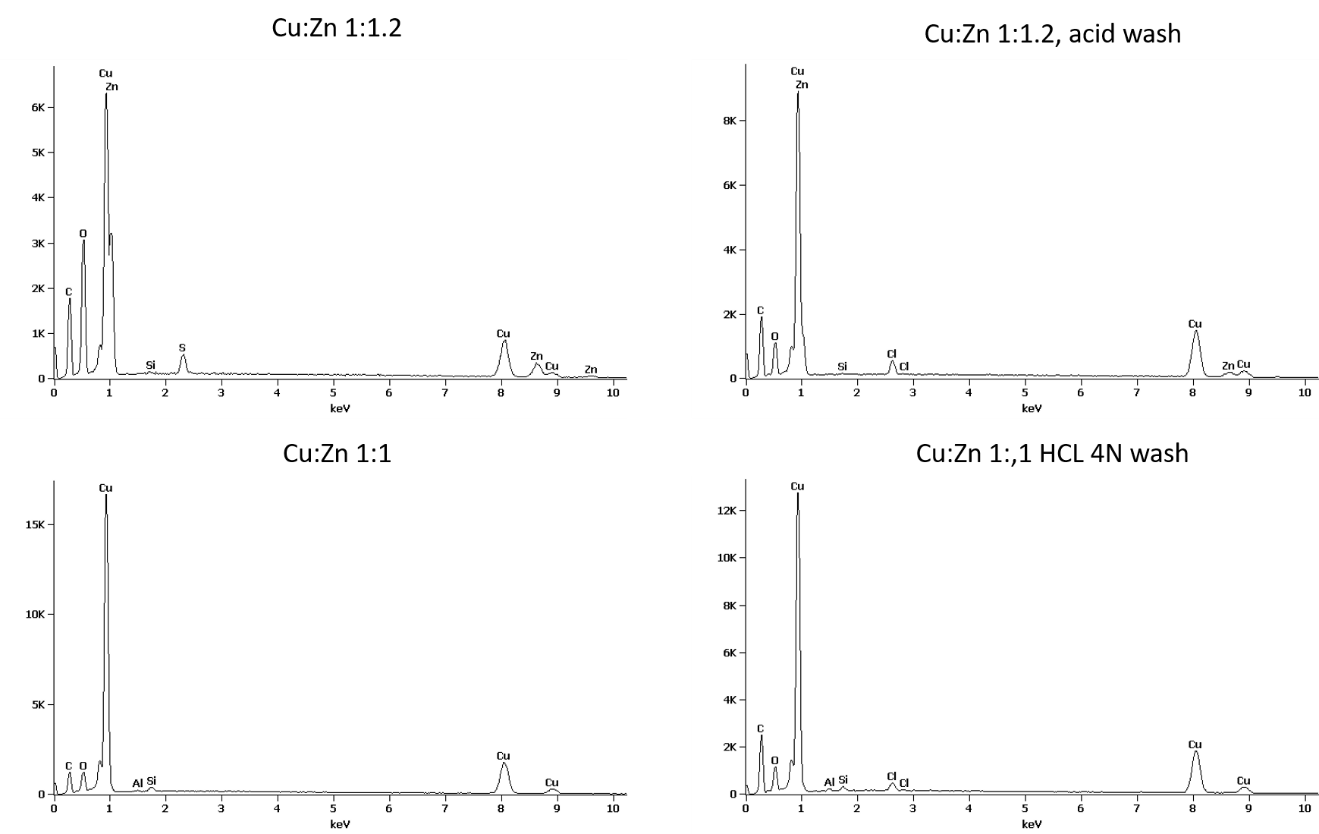


**Figure SI2**: EDAX analysis of copper reduced by zinc at a ratio of 1:1 or 1:1.2 and with or without a 4N HCl acid wash. Decreasing the molar ratio and washing with HCl reduces the presence of zinc in the composites.

**Figure SI3:** X-ray diffraction (XRD) results of composites:

**[Cu]**

**LK20-mer@Cu**

**LK10-mer@Cu**

**Table SI1:** ICP results of three independent repeats. Ratio of copper ions released from [Cu] to copper ions released from LK20:mer@[Cu].

|  | [Cu] : LK20-mer@[Cu] |
| --- | --- |
| 1 | 1:1 |
| 2 | 0.94:1 |
| 3 | 0.85:1 |

**Figure SI4:** Thermal gravity analysis of composites and first derivative.


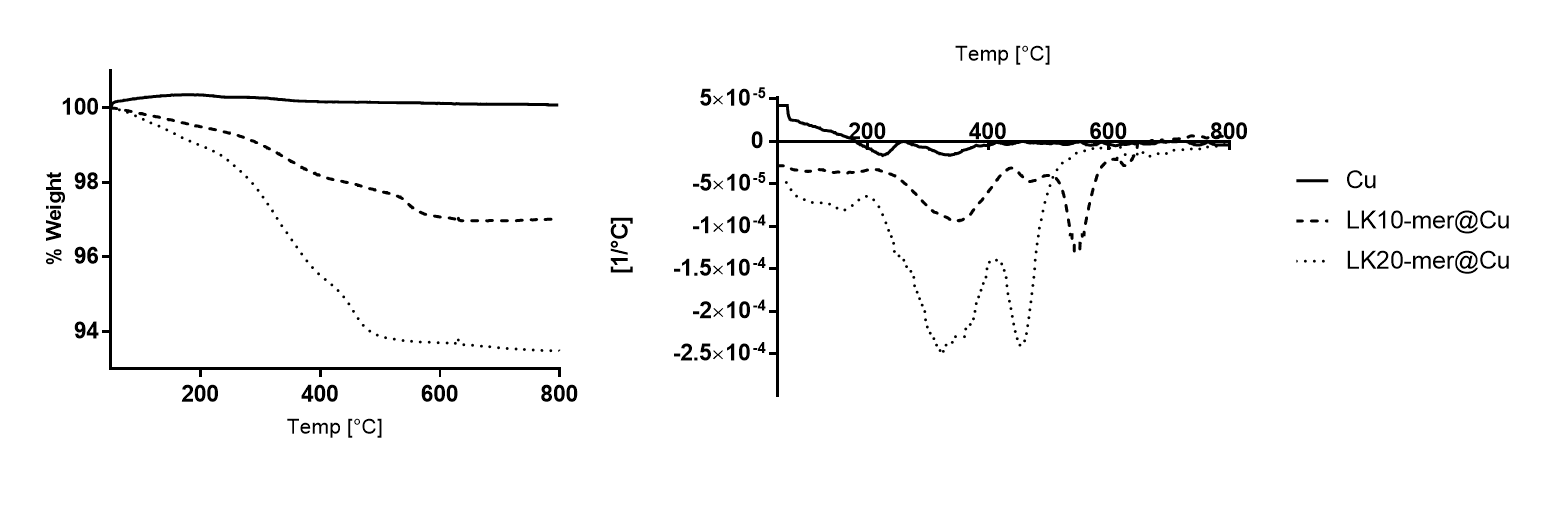


**Table SI2:** Examination of peptide release. Organic elemental analysis of composites before and after incubation in water, presented as percentage of weight.

|  | **N** | | **C** | | **H** | | **S** | |
| --- | --- | --- | --- | --- | --- | --- | --- | --- |
|  | AVG | SE | AVG | SE | AVG | SE | AVG | SE |
| **[Cu]** | 0.0026 | 0.0022 | 0.0331 | 0.0029 | 0.0000 | 0.0000 | 0.0037 | 0.0032 |
| **[Cu] released** | 0.0118 | 0.0072 | 0.0770 | 0.0138 | 0.0000 | 0.0000 | 0.0222 | 0.0124 |
| **LK10@[Cu]** | 0.1231 | 0.0102 | 0.7446 | 0.0028 | 0.0593 | 0.0298 | 0.1007 | 0.0330 |
| **LK10@[Cu] released** | 0.1156 | 0.0117 | 0.9092 | 0.0080 | 0.0630 | 0.0315 | 0.0897 | 0.0450 |
| **LK20@[Cu]** | 0.2277 | 0.0117 | 1.1329 | 0.0357 | 0.2025 | 0.0064 | 0.1562 | 0.0439 |
| **LK20@[Cu] released** | 0.1031 | 0.0035 | 1.2962 | 0.0251 | 0.1069 | 0.0535 | 0.0879 | 0.0256 |

_*Two independent experiments, 2 technical repeats for each._
